# 1,7,7-Trimethyl-3-(naphthalen-2-ylcarbon­yl)bi­cyclo­[2.2.1]heptan-2-one

**DOI:** 10.1107/S2414314620016624

**Published:** 2020-12-24

**Authors:** Sabrina Bendia, Kamel Ouari, Mustapha Ait Ali, Larbi el Firdousi, Alexander Y. Nazarenko

**Affiliations:** a Laboratoire d’Electrochimie, d’Ingénierie Moléculaire et de Catalyse Rédox (LEIMCR), Faculté de Technologie, Université Ferhat Abbas-Sétif-1, Sétif, 19000 , Algeria; b Université Cadi Ayyad Faculté des Sciences Semlalia, Departement de Chimie, BP 2390, 40001, Marrakech, Morocco; c Chemistry Department, State University of New York, College at Buffalo, 1300 Elmwood Ave, Buffalo, NY 14222-1095, USA; Howard University, USA

**Keywords:** crystal structure, β-diketone, naphth­yl, camphor

## Abstract

The title compound, C_21_H_22_O_2_, crystallizes in its keto form. The mol­ecules are connected *via* weak C—H⋯O inter­actions, forming infinite chains perpendic­ular to the [001] axis.

## Abstract 2

The title compound, C_21_H_22_O_2_, crystallizes in its keto-form. The mol­ecules are connected *via* weak C—H⋯O inter­actions, forming infinite chains perpendicular to the [001] axis.

## Graphical abstract

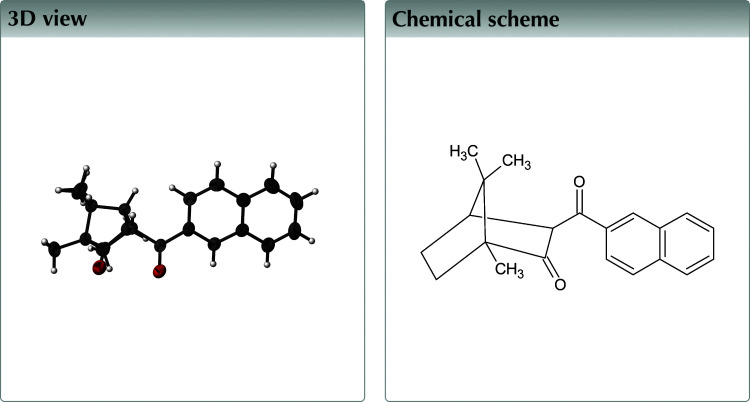

## Structure description

Chiral β-diketonato ligands are used in catalysis and spectroscopy; chiral naphthyl derivatives have recently been prepared (Clark *et al.*, 2013 ▸). However, no naphthyl-substituted β-diketones were found in the Cambridge Structure Database (Groom *et al.*, 2016 ▸) in November 2020.

**Figure 1:**
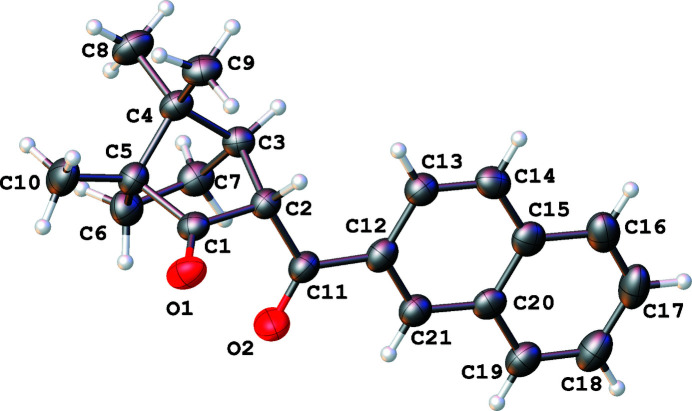
Numbering scheme of the title compound with 50% probability displacement ellipsoids.

The title compound, C_21_H_22_O_2_, crystallizes in its keto-form (Fig. 1 ▸). The shape of both the camphor and naphthyl fragments is essentially the same as in their parent mol­ecules.

**Figure 2:**
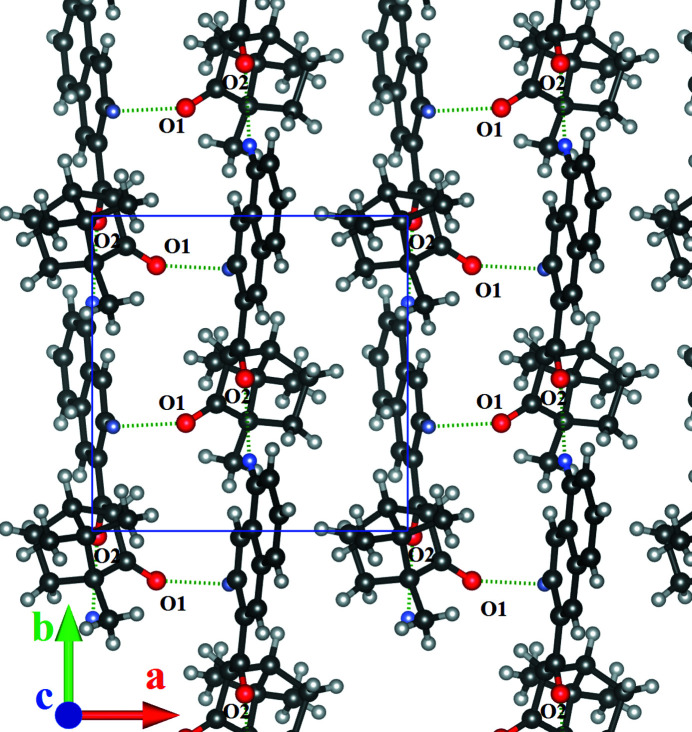
Packing diagram of the title compound showing C—H⋯O hydrogen bonding; the view along the [001] vector, which is parallel to the **4_1_** screw axis. Only one layer of mol­ecules is shown; the screw-axis symmetry operation rotates each subsequent layer by 90° and moves by *c*/4.

There are no strong inter­molecular inter­actions in this structure. The mol­ecules are connected *via* weak C—H⋯O bonds (Table 1 ▸), forming infinite chains perpendicular to the [001] axis (Fig. 2 ▸). The hydrogen atoms of the naphthyl ring system and atoms H9*B*, H9*C*, and H10*B* of the methyl groups of the camphor fragment help to assemble these chains in the crystal *via* van der Waals inter­actions.

## Synthesis and crystallization

The title compound was prepared by a procedure reported earlier (Clark *et al.*, 2013 ▸) and was purified by recrystallization from hexane solution (m.p. 399 K). Elemental analysis for C_21_H_22_O_2_, calculated C 82.30, H 7.24; found C 82.23, H 7.15. ^1^H NMR: (DMSO-*d*_6_, δ p.p.m.): 12.50 (*s*, OH), 9.00–7.40 (*m*, H_Ar_), 2.80–0.80 (*m*, H_Camphor_); ^13^C NMR: (DMSO-*d*_6_, δ p.p.m.): 212–194 (C=O), 135–115 (C_Ar_), 64–9 (C_Camphor_). UV–vis in aceto­nitrile (λ_max_ (nm), [ɛ] (l mol^−1^ cm^−1^)): 272 [10197], 283 [12668], 292 [10352], 325 [8294]. Single crystals were grown by slow evaporation of a methanol solution at room temperature.

## Refinement

Crystal data, data collection and structure refinement details are summarized in Table 2 ▸.

## Supplementary Material

Crystal structure: contains datablock(s) I. DOI: 10.1107/S2414314620016624/bv4034sup1.cif

Structure factors: contains datablock(s) I. DOI: 10.1107/S2414314620016624/bv4034Isup2.hkl

Supporting information file. DOI: 10.1107/S2414314620016624/bv4034Isup3.cdx

Supporting information file. DOI: 10.1107/S2414314620016624/bv4034Isup4.cml

CCDC reference: 2052026

Additional supporting information:  crystallographic information; 3D view; checkCIF report

## Figures and Tables

**Table 1:** Hydrogen-bond geometry (Å, °)

*D*—H⋯*A*	*D*—H	H⋯*A*	*D*⋯*A*	*D*—H⋯*A*
C13—H13⋯O1^i^	0.95	2.57	3.296 (3)	134
C16—H16⋯O2^ii^	0.95	2.57	3.458 (3)	156

Symmetry codes: (i) 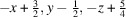 ; (ii) 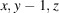 .

**Table 2:** Experimental details

Crystal data	
Chemical formula	C_21_H_22_O_2_
*M*_r_	306.38
Crystal system, space group	Tetragonal, *P*4_1_2_1_2
Temperature (K)	173
*a*, *c* (Å)	9.5637 (3), 36.3395 (10)
*V* (Å^3^)	3323.8 (2)
*Z*	8
Radiation type	Cu *K*α
μ (mm^−1^)	0.60
Crystal size (mm)	0.41 × 0.33 × 0.17

Data collection	
Diffractometer	Bruker PHOTON-100 CMOS
Absorption correction	Multi-scan (*SADABS*; Krause *et al.*, 2015 ▸)
*T*_min_, *T*_max_	0.922, 1.000
No. of measured, independent and observed [*I* > 2σ(*I*)] reflections	27362, 3363, 3123
*R*_int_	0.034
(sin θ/λ)_max_ (Å^−1^)	0.627

Refinement	
*R*[*F* ^2^ > 2σ(*F* ^2^)], *wR*(*F* ^2^), *S*	0.042, 0.110, 1.05
No. of reflections	3363
No. of parameters	211
H-atom treatment	H-atom parameters constrained
Δρ_max_, Δρ_min_ (e Å^−3^)	0.33, −0.16
Absolute structure	Flack *x* determined using 1176 quotients [(*I* ^+^)−(*I* ^−^)]/[(*I* ^+^)+(*I* ^−^)] (Parsons et al., 2013 ▸)
Absolute structure parameter	−0.04 (10)

Computer programs: *APEX2* and *SAINT* (Bruker, 2016 ▸), *SHELXT* (Sheldrick, 2015*a* ▸), *SHELXL* (Sheldrick, 2015*b* ▸), *OLEX2* (Dolomanov *et al.*, 2009 ▸) and *VESTA* (Momma & Izumi, 2011 ▸).

## full crystallographic data

### Crystal data

**Table:**

C_21_H_22_O_2_	Melting point: 399 K
*M**_r_* = 306.38	Cu *K*α radiation, λ = 1.54178 Å
Tetragonal, *P*4_1_2_1_2	Cell parameters from 9853 reflections
*a* = 9.5637 (3) Å	θ = 4.6–74.7°
*c* = 36.3395 (10) Å	µ = 0.60 mm^−^^1^
*V* = 3323.8 (2) Å^3^	*T* = 173 K
*Z* = 8	Block, colourless
*F*(000) = 1312	0.41 × 0.33 × 0.17 mm
*D*_x_ = 1.225 Mg m^−^^3^	

### Data collection

**Table:**

Bruker PHOTON-100 CMOS diffractometer	3363 independent reflections
Radiation source: sealedtube	3123 reflections with *I* > 2σ(*I*)
Detector resolution: 10.8 pixels mm^-1^	*R*_int_ = 0.034
φ and ω scans	θ_max_ = 75.2°, θ_min_ = 4.8°
Absorption correction: multi-scan (SADABS; Krause *et al.*, 2015)	*h* = −11→9
*T*_min_ = 0.922, *T*_max_ = 1.000	*k* = −11→10
27362 measured reflections	*l* = −43→45

### Refinement

**Table:**

Refinement on *F*^2^	Secondary atom site location: difference Fourier map
Least-squares matrix: full	Hydrogen site location: inferred from neighbouring sites
*R*[*F*^2^ > 2σ(*F*^2^)] = 0.042	H-atom parameters constrained
*wR*(*F*^2^) = 0.110	*w* = 1/[σ^2^(*F*_o_^2^) + (0.0568*P*)^2^ + 0.9139*P*] where *P* = (*F*_o_^2^ + 2*F*_c_^2^)/3
*S* = 1.05	(Δ/σ)_max_ = 0.001
3363 reflections	Δρ_max_ = 0.33 e Å^−^^3^
211 parameters	Δρ_min_ = −0.16 e Å^−^^3^
0 restraints	Absolute structure: Flack *x* determined using 1176 quotients [(*I*^+^)-(*I*^-^)]/[(*I*^+^)+(*I*^-^)] (Parsons et al., 2013)
Primary atom site location: dual	Absolute structure parameter: −0.04 (10)

### Special details

**Table:**

Geometry. All esds (except the esd in the dihedral angle between two l.s. planes) are estimated using the full covariance matrix. The cell esds are taken into account individually in the estimation of esds in distances, angles and torsion angles; correlations between esds in cell parameters are only used when they are defined by crystal symmetry. An approximate (isotropic) treatment of cell esds is used for estimating esds involving l.s. planes.

### Fractional atomic coordinates and isotropic or equivalent isotropic displacement parameters (Å^2^)

**Table:**

	*x*	*y*	*z*	*U*_iso_*/*U*_eq_	
O1	0.70347 (19)	0.6575 (2)	0.64361 (5)	0.0464 (4)	
O2	0.5159 (2)	0.51669 (19)	0.69923 (4)	0.0496 (5)	
C1	0.6066 (2)	0.5955 (2)	0.62993 (5)	0.0332 (5)	
C12	0.5164 (2)	0.2695 (3)	0.69118 (6)	0.0365 (5)	
C20	0.4639 (2)	0.1076 (3)	0.74128 (6)	0.0352 (5)	
C2	0.5645 (2)	0.4436 (2)	0.63805 (6)	0.0322 (5)	
H2	0.641392	0.379430	0.630123	0.039*	
C11	0.5318 (2)	0.4187 (2)	0.67846 (6)	0.0361 (5)	
C4	0.4804 (2)	0.5150 (2)	0.57811 (6)	0.0353 (5)	
C21	0.4779 (2)	0.2461 (3)	0.72708 (6)	0.0377 (5)	
H21	0.460193	0.323627	0.742761	0.045*	
C5	0.5053 (2)	0.6502 (2)	0.60124 (6)	0.0367 (5)	
C3	0.4366 (2)	0.4255 (2)	0.61188 (6)	0.0332 (5)	
H3	0.413105	0.326134	0.605939	0.040*	
C7	0.3144 (2)	0.5104 (3)	0.62806 (6)	0.0409 (5)	
H7A	0.299828	0.488296	0.654394	0.049*	
H7B	0.226558	0.492847	0.614409	0.049*	
C15	0.4932 (2)	−0.0064 (3)	0.71753 (6)	0.0394 (5)	
C13	0.5416 (2)	0.1533 (3)	0.66742 (6)	0.0387 (5)	
H13	0.565984	0.168661	0.642397	0.046*	
C14	0.5307 (2)	0.0201 (3)	0.68070 (7)	0.0408 (5)	
H14	0.548878	−0.056467	0.664725	0.049*	
C8	0.3638 (3)	0.5307 (3)	0.54932 (7)	0.0499 (6)	
H8A	0.282051	0.574768	0.560714	0.075*	
H8B	0.337812	0.438197	0.539925	0.075*	
H8C	0.397117	0.589021	0.528961	0.075*	
C9	0.6120 (3)	0.4631 (3)	0.55838 (6)	0.0426 (6)	
H9A	0.640885	0.532133	0.539957	0.064*	
H9B	0.591951	0.373884	0.546203	0.064*	
H9C	0.687276	0.450052	0.576340	0.064*	
C19	0.4206 (3)	0.0829 (3)	0.77769 (7)	0.0438 (6)	
H19	0.400420	0.159300	0.793543	0.053*	
C16	0.4805 (3)	−0.1453 (3)	0.73169 (8)	0.0496 (6)	
H16	0.501686	−0.223071	0.716425	0.060*	
C6	0.3646 (3)	0.6628 (3)	0.62289 (7)	0.0442 (6)	
H6A	0.379542	0.708616	0.647000	0.053*	
H6B	0.295274	0.717616	0.608703	0.053*	
C10	0.5525 (3)	0.7813 (3)	0.58164 (7)	0.0523 (7)	
H10A	0.565896	0.856496	0.599628	0.078*	
H10B	0.481338	0.809412	0.563703	0.078*	
H10C	0.640935	0.763122	0.568879	0.078*	
C17	0.4383 (3)	−0.1660 (3)	0.76687 (8)	0.0537 (7)	
H17	0.429241	−0.258625	0.776009	0.064*	
C18	0.4076 (3)	−0.0513 (3)	0.79021 (7)	0.0513 (7)	
H18	0.377741	−0.067775	0.814759	0.062*	

### Atomic displacement parameters (Å^2^)

**Table:**

	*U*^11^	*U*^22^	*U*^33^	*U*^12^	*U*^13^	*U*^23^
O1	0.0456 (10)	0.0561 (11)	0.0375 (8)	−0.0160 (8)	−0.0059 (7)	0.0023 (8)
O2	0.0678 (12)	0.0467 (10)	0.0343 (8)	0.0024 (8)	0.0081 (8)	0.0006 (7)
C1	0.0344 (11)	0.0380 (12)	0.0273 (9)	−0.0036 (9)	0.0034 (8)	0.0012 (8)
C12	0.0288 (11)	0.0459 (13)	0.0350 (10)	0.0010 (9)	−0.0009 (9)	0.0071 (9)
C20	0.0255 (10)	0.0455 (13)	0.0345 (10)	−0.0012 (9)	−0.0056 (8)	0.0008 (9)
C2	0.0295 (10)	0.0364 (11)	0.0308 (10)	0.0007 (9)	0.0018 (8)	0.0020 (8)
C11	0.0370 (12)	0.0401 (12)	0.0311 (10)	0.0025 (9)	0.0002 (9)	0.0040 (9)
C4	0.0310 (11)	0.0442 (13)	0.0306 (10)	−0.0031 (9)	−0.0019 (8)	0.0020 (9)
C21	0.0352 (11)	0.0427 (13)	0.0353 (10)	0.0021 (10)	−0.0015 (9)	0.0014 (10)
C5	0.0369 (12)	0.0385 (12)	0.0347 (10)	0.0005 (10)	−0.0007 (9)	0.0052 (9)
C3	0.0290 (11)	0.0370 (11)	0.0337 (10)	−0.0032 (9)	0.0015 (8)	−0.0005 (9)
C7	0.0301 (11)	0.0519 (15)	0.0406 (11)	0.0001 (10)	0.0035 (9)	0.0008 (10)
C15	0.0283 (11)	0.0460 (13)	0.0439 (11)	0.0014 (9)	−0.0072 (9)	0.0020 (10)
C13	0.0353 (12)	0.0439 (13)	0.0370 (11)	0.0023 (10)	0.0012 (9)	0.0007 (10)
C14	0.0351 (12)	0.0441 (13)	0.0432 (11)	0.0052 (10)	−0.0010 (10)	−0.0066 (10)
C8	0.0410 (14)	0.0705 (18)	0.0381 (12)	−0.0032 (13)	−0.0088 (10)	0.0036 (12)
C9	0.0380 (12)	0.0572 (15)	0.0324 (10)	−0.0050 (11)	0.0045 (9)	−0.0035 (10)
C19	0.0396 (13)	0.0527 (15)	0.0390 (11)	−0.0023 (11)	−0.0045 (10)	0.0055 (11)
C16	0.0442 (14)	0.0422 (14)	0.0625 (16)	−0.0025 (11)	−0.0117 (12)	0.0047 (12)
C6	0.0416 (13)	0.0442 (13)	0.0467 (13)	0.0101 (11)	0.0013 (11)	0.0008 (11)
C10	0.0639 (18)	0.0459 (15)	0.0471 (13)	−0.0080 (13)	−0.0071 (12)	0.0129 (11)
C17	0.0502 (16)	0.0447 (14)	0.0662 (16)	−0.0085 (12)	−0.0135 (13)	0.0198 (13)
C18	0.0438 (14)	0.0618 (17)	0.0482 (13)	−0.0051 (12)	−0.0043 (11)	0.0181 (12)

### Geometric parameters (Å, º)

**Table:**

O1—C1	1.207 (3)	C7—C6	1.545 (4)
O2—C11	1.213 (3)	C15—C14	1.409 (3)
C1—C2	1.535 (3)	C15—C16	1.430 (4)
C1—C5	1.516 (3)	C13—H13	0.9500
C12—C11	1.507 (3)	C13—C14	1.367 (4)
C12—C21	1.374 (3)	C14—H14	0.9500
C12—C13	1.428 (3)	C8—H8A	0.9800
C20—C21	1.428 (3)	C8—H8B	0.9800
C20—C15	1.418 (3)	C8—H8C	0.9800
C20—C19	1.406 (3)	C9—H9A	0.9800
C2—H2	1.0000	C9—H9B	0.9800
C2—C11	1.521 (3)	C9—H9C	0.9800
C2—C3	1.559 (3)	C19—H19	0.9500
C4—C5	1.560 (3)	C19—C18	1.367 (4)
C4—C3	1.554 (3)	C16—H16	0.9500
C4—C8	1.537 (3)	C16—C17	1.355 (4)
C4—C9	1.531 (3)	C6—H6A	0.9900
C21—H21	0.9500	C6—H6B	0.9900
C5—C6	1.564 (3)	C10—H10A	0.9800
C5—C10	1.511 (3)	C10—H10B	0.9800
C3—H3	1.0000	C10—H10C	0.9800
C3—C7	1.540 (3)	C17—H17	0.9500
C7—H7A	0.9900	C17—C18	1.418 (4)
C7—H7B	0.9900	C18—H18	0.9500
			
O1—C1—C2	125.9 (2)	C20—C15—C16	118.6 (2)
O1—C1—C5	127.1 (2)	C14—C15—C20	119.4 (2)
C5—C1—C2	106.96 (18)	C14—C15—C16	122.0 (2)
C21—C12—C11	118.1 (2)	C12—C13—H13	120.0
C21—C12—C13	119.5 (2)	C14—C13—C12	120.0 (2)
C13—C12—C11	122.31 (19)	C14—C13—H13	120.0
C15—C20—C21	118.3 (2)	C15—C14—H14	119.3
C19—C20—C21	121.5 (2)	C13—C14—C15	121.5 (2)
C19—C20—C15	120.1 (2)	C13—C14—H14	119.3
C1—C2—H2	109.5	C4—C8—H8A	109.5
C1—C2—C3	101.15 (16)	C4—C8—H8B	109.5
C11—C2—C1	112.83 (18)	C4—C8—H8C	109.5
C11—C2—H2	109.5	H8A—C8—H8B	109.5
C11—C2—C3	114.20 (17)	H8A—C8—H8C	109.5
C3—C2—H2	109.5	H8B—C8—H8C	109.5
O2—C11—C12	121.90 (19)	C4—C9—H9A	109.5
O2—C11—C2	120.4 (2)	C4—C9—H9B	109.5
C12—C11—C2	117.72 (19)	C4—C9—H9C	109.5
C3—C4—C5	94.14 (16)	H9A—C9—H9B	109.5
C8—C4—C5	113.4 (2)	H9A—C9—H9C	109.5
C8—C4—C3	113.29 (19)	H9B—C9—H9C	109.5
C9—C4—C5	113.31 (19)	C20—C19—H19	120.1
C9—C4—C3	114.39 (19)	C18—C19—C20	119.8 (3)
C9—C4—C8	108.02 (19)	C18—C19—H19	120.1
C12—C21—C20	121.3 (2)	C15—C16—H16	120.0
C12—C21—H21	119.4	C17—C16—C15	120.1 (3)
C20—C21—H21	119.4	C17—C16—H16	120.0
C1—C5—C4	100.46 (18)	C5—C6—H6A	110.8
C1—C5—C6	103.31 (18)	C5—C6—H6B	110.8
C4—C5—C6	101.74 (18)	C7—C6—C5	104.83 (19)
C10—C5—C1	114.8 (2)	C7—C6—H6A	110.8
C10—C5—C4	118.64 (19)	C7—C6—H6B	110.8
C10—C5—C6	115.5 (2)	H6A—C6—H6B	108.9
C2—C3—H3	114.4	C5—C10—H10A	109.5
C4—C3—C2	102.03 (16)	C5—C10—H10B	109.5
C4—C3—H3	114.4	C5—C10—H10C	109.5
C7—C3—C2	107.70 (17)	H10A—C10—H10B	109.5
C7—C3—C4	102.45 (18)	H10A—C10—H10C	109.5
C7—C3—H3	114.4	H10B—C10—H10C	109.5
C3—C7—H7A	111.3	C16—C17—H17	119.6
C3—C7—H7B	111.3	C16—C17—C18	120.8 (3)
C3—C7—C6	102.44 (18)	C18—C17—H17	119.6
H7A—C7—H7B	109.2	C19—C18—C17	120.6 (2)
C6—C7—H7A	111.3	C19—C18—H18	119.7
C6—C7—H7B	111.3	C17—C18—H18	119.7
			
O1—C1—C2—C11	58.5 (3)	C5—C4—C3—C2	−55.30 (19)
O1—C1—C2—C3	−179.1 (2)	C5—C4—C3—C7	56.14 (19)
O1—C1—C5—C4	144.2 (2)	C3—C2—C11—O2	−101.7 (3)
O1—C1—C5—C6	−110.9 (3)	C3—C2—C11—C12	76.7 (3)
O1—C1—C5—C10	15.7 (3)	C3—C4—C5—C1	54.17 (19)
C1—C2—C11—O2	13.1 (3)	C3—C4—C5—C6	−51.94 (19)
C1—C2—C11—C12	−168.51 (19)	C3—C4—C5—C10	−179.9 (2)
C1—C2—C3—C4	35.2 (2)	C3—C7—C6—C5	4.6 (2)
C1—C2—C3—C7	−72.2 (2)	C15—C20—C21—C12	1.1 (3)
C1—C5—C6—C7	−73.3 (2)	C15—C20—C19—C18	0.4 (3)
C12—C13—C14—C15	0.8 (4)	C15—C16—C17—C18	−0.6 (4)
C20—C15—C14—C13	1.0 (3)	C13—C12—C11—O2	−176.8 (2)
C20—C15—C16—C17	1.4 (4)	C13—C12—C11—C2	4.8 (3)
C20—C19—C18—C17	0.4 (4)	C13—C12—C21—C20	0.6 (3)
C2—C1—C5—C4	−34.7 (2)	C14—C15—C16—C17	−177.2 (2)
C2—C1—C5—C6	70.1 (2)	C8—C4—C5—C1	171.77 (19)
C2—C1—C5—C10	−163.2 (2)	C8—C4—C5—C6	65.7 (2)
C2—C3—C7—C6	68.6 (2)	C8—C4—C5—C10	−62.3 (3)
C11—C12—C21—C20	−178.8 (2)	C8—C4—C3—C2	−172.96 (19)
C11—C12—C13—C14	177.8 (2)	C8—C4—C3—C7	−61.5 (2)
C11—C2—C3—C4	156.71 (18)	C9—C4—C5—C1	−64.6 (2)
C11—C2—C3—C7	49.3 (2)	C9—C4—C5—C6	−170.76 (18)
C4—C5—C6—C7	30.5 (2)	C9—C4—C5—C10	61.3 (3)
C4—C3—C7—C6	−38.6 (2)	C9—C4—C3—C2	62.6 (2)
C21—C12—C11—O2	2.6 (3)	C9—C4—C3—C7	174.07 (19)
C21—C12—C11—C2	−175.8 (2)	C19—C20—C21—C12	−178.1 (2)
C21—C12—C13—C14	−1.6 (3)	C19—C20—C15—C14	177.3 (2)
C21—C20—C15—C14	−1.9 (3)	C19—C20—C15—C16	−1.3 (3)
C21—C20—C15—C16	179.5 (2)	C16—C15—C14—C13	179.6 (2)
C21—C20—C19—C18	179.6 (2)	C16—C17—C18—C19	−0.3 (4)
C5—C1—C2—C11	−122.51 (19)	C10—C5—C6—C7	160.4 (2)
C5—C1—C2—C3	−0.1 (2)		

### Hydrogen-bond geometry (Å, º)

**Table:**

*D*—H···*A*	*D*—H	H···*A*	*D*···*A*	*D*—H···*A*
C13—H13···O1^i^	0.95	2.57	3.296 (3)	134
C16—H16···O2^ii^	0.95	2.57	3.458 (3)	156

Symmetry codes: (i) −*x*+3/2, *y*−1/2, −*z*+5/4; (ii) *x*, *y*−1, *z*.
